# Monoclonal Gammopathy of Undetermined Significance (MGUS) in a Man with Fragile X-associated Tremor/Ataxia Syndrome 

**DOI:** 10.1155/2011/143132

**Published:** 2011-12-01

**Authors:** Tanjung A. Sumekar, Aneel A. Ashrani, Tri I. Winarni, Randi J. Hagerman

**Affiliations:** ^1^Medical Investigation of Neurodevelopmental Disorders (MIND) Institute, Davis Medical Center, University of California, 2825 50th Street, Sacramento, CA 95817, USA; ^2^Division of Human Genetics, Center for Biomedical Research, Faculty of Medicine, Diponegoro University, Jalan Dr. Sutomo No. 14, Semarang 50321, Indonesia; ^3^Division of Hematology, Department of Internal Medicine, College of Medicine, Mayo Clinic, 200 First Street SW, Rochester, MN 55905, USA; ^4^Department of Pediatrics, Davis Medical Center, University of California, Sacramento, CA 95817, USA

## Abstract

We report the clinical presentation and laboratory findings of a 69-year-old man with fragile X-associated tremor ataxia syndrome (FXTAS), a progressive neurodegenerative disorder, who was noted to have monoclonal gammopathy of undetermined significance (MGUS), a plasma cell proliferative disorder and a precursor disease of multiple myeloma. Both MGUS and FXTAS are associated with microRNA (miRNA) dysregulation. We speculate that individuals with FXTAS may be predisposed to MGUS and further studies are warranted regarding this association.

## 1. Introduction

Fragile X-associated tremor/ataxia syndrome (FXTAS) is an adult progressive neurodegenerative disorder that affects predominantly male carriers of the fragile X mental retardation 1 (*FMR1*) gene premutation (CGG expansion of 55–200 repeats). The clinical features of FXTAS are progressive intention tremor, cerebellar ataxia, autonomic dysfunction, peripheral neuropathy, cognitive decline, middle cerebellar peduncle signs (MCPs), and white matter disease combined with brain atrophy on MRI [1–4].

The penetrance of FXTAS in male carriers older than age 50 is approximately 30 to 45.5% and 8 to 16.5% in females [2, 5, 6]. Overproduction of the *FMR1* mRNA, up to 8 fold times normal, is the basic mechanism of FXTAS [7]. The elevated *FMR1* mRNA causes a gain-of-function toxicity to cells leading to the formation of intranuclear inclusions in neurons and astrocytes throughout the central nervous system (CNS) and subsequent sequestration of critical proteins necessary for normal cell function [8, 9]. Mitochondrial dysfunction also occurs in older premutation carriers both with and without FXTAS [10, 11]. This cellular dysfunction leads to neuronal cell death and white matter disease in the CNS [3, 12, 13] associated with the clinical features of FXTAS.

Monoclonal gammopathy of undetermined significance (MGUS) is a plasma cell proliferative disorder characterized by a plasma cell level of less than 10% in the bone marrow, a monoclonal paraprotein band less than 30 g/L, and no end organ damage (CRAB—hypercalcemia, renal insufficiency, anemia, and bone lesions) [14]. It is an age-related medical condition found predominantly in older persons [15]. The diagnosis of MGUS is usually an unexpected event during an evaluation, and because it is typically asymptomatic, it is underdiagnosed [16]. However, some abnormalities such as bone loss or osteoporosis, peripheral neuropathy, and thromboembolic events can be associated to this disorder [17]. Individuals with MGUS have a higher risk of developing multiple myeloma (MM) or related malignancy [18–20]. Race, ethinicity, advance age, male sex [15], family history [21], and exposure to certain pesticides [22] increase the risk of MGUS indicating that MGUS is a multifactorial condition.

The pathogenesis of MGUS is hypothesized to begin with an abnormal response to antigenic stimulation mediated by toll-like receptors (TLRs) and interleukin IL-6 receptors and IL-1*β* [23, 24]. This results in cytogenetic abnormalities—either hyperdiploidy or immunoglobulin heavy chain (IgH) translocations [25]. When it is followed by the second hit including RAS or p53 mutation, p18 deletion, myc dysregulation, MGUS progression to MM then occurs [26].

Dysregulation of microRNAs (miRNAs), the small noncoding RNAs involved in posttranscriptional gene regulation, plays a role in MGUS progression to MM [27]. MiRNAs have been demonstrated to play a role in regulation of apoptosis, proliferation, differentiation, cell survival, and oncogenesis [28]. Abnormal miRNA regulation may lead to oncogenesis, particularly MM [29–31]. The miRNA profiles discrepantly expressed in MGUS also show similar aberrant expression in MM suggesting the role of miRNAs in early myelomagenesis [27].

MiRNAs are encoded throughout the genome and are transcribed into pri-miRNA. The pri-miRNA is cleaved in the nucleus by the Drosha RNase III endonuclease. Pre-miRNAs are transported to the cytoplasm. In cytoplasm, Dicer, a ribonuclease III, cleaves the pre-miRNA into the mature miRNA which forms a complex with RNA-induced silencing complex (RISC). This complex directs the miRNA to the target mRNA leading either to degradation of target mRNA or translational repression [32]. In FXTAS patients, there is Drosha/DGCR8 sequestration by the elevated *FMR1* mRNAs that leads to global miRNA dysregulation [33]. We speculate that this global miRNA dysregulation may predispose individuals to MGUS. Here we report the clinical presentation and laboratory findings of a man with FXTAS and MGUS.

## 2. Clinical Report

A 69-year-old Caucasian man with FXTAS had a premutation of 98 to 103 CGG repeats and an *FMR1* mRNA level of 2.33 ± 0.02 times normal. His intention tremor began at age 55 and has been progressive. Currently, it interferes with his activities of daily living (ADL) including eating and handwriting. His gait imbalance began at age 58 and he started using a walker at age 66. His short-term memory problems began at age 63. His stamina has decreased significantly over the last few years and he has some decreased sensation in his lower legs in addition to pain with walking. He has autonomic dysfunction including bladder incontinence and erectile dysfunction beginning at age 66. His past medical history includes hypertension, type 2 diabetes, hyperlipidemia, hypothyroidism, sleep apnea, and migraine headaches (Table 1).

His family history includes a carrier daughter with fragile X-associated primary ovarian insufficiency (FXPOI). His brother has had severe FXTAS with two surgeries for deep brain stimulation. His mother, a carrier, has also had tremor, cognitive decline, and non-Hodgkin's lymphoma and it is likely that she also has FXTAS.

On physical examination, his blood pressure was 167/62 mmHg, the heart rate was 71 bpm, the head circumference was 59.5 cm, his height was 172.7 cm, and his weight was 87.5 kg. The ears were slightly prominent. His neurological examination was abnormal with a severe intention tremor with finger to nose touching but absent at rest. There were no deep tendon reflexes but positive primitive reflexes including the snout reflex. His vibration sense was decreased in both feet. His gait was ataxic and broad based. He cannot tandem walk and he had a positive pull test. On MRI, he had significant brain atrophy and white matter disease including a positive MCP sign.

As a part of a workup for his mild normocytic anemia, serum protein electrophoresis with immunofixation revealed 1 g/dL IgG kappa monoclonal paraproteinemia. His serum creatinine was mildly elevated at 1.3 mg/dL with estimated GFR of 55 mL/min. Calcium and highly sensitive C-reactive protein were within the normal reference range. The quantitative immunoglobulins revealed normal IgG and IgA levels with decreased IgM (17 mg/dL; lower reference range 37 mg/dL). The free kappa light chain is elevated at 7.81 mg/dL (upper reference range 1.92 mg/dL) with normal lambda light chain (1.88 mg/dL). The kappa to lambda light chain ratio was elevated at 4.15 (upper reference range 1.65). The beta-2 microglobulin is elevated at 3.38 mcg/mL (upper reference range 1.8 mcg/mL). The skeletal survey did not reveal any lytic or sclerotic lesions. The bone marrow biopsy revealed a normocellular marrow (30% cellularity) with less than 5% kappa light chain restricted plasma cells (Table 2). 

## 3. Discussion

This is the first clinical report of MGUS in an individual with FXTAS. Although MGUS is an asymptomatic condition, it is associated with peripheral neuropathy, osteoporosis, and thromboembolic events. Symptoms shared by FXTAS and MGUS include peripheral neuropathy. His risk factors for developing an MGUS included advancing age, male sex, and having a mother with non-Hodgkin's lymphoma, a hematologic cancer. We also suggest that those with FXTAS have a greater risk for MGUS through the common finding of miRNA dysregulation in both disorders.

The DROSHA/DGCR8 sequestration mechanism in FXTAS leads to miRNA alteration, specifically downregulation of several miRNAs [33]. Thus, it may play a role in oncogenesis and tumor biology through posttrancriptional gene regulation impacting the mechanism of proliferation, differentiation, apoptosis, metastasis, and cell survival [28]. In MGUS, miRNAs play an important role in early changes associated with the development of the abnormal clonal plasma cell and have a potential role in altering the p53 pathway [29]. Downregulation or absence of miRNAs has been reported in many cancers [34], including other hemathologic malignancies such as acute myeloid leukemia [35], chronic lymphocytic leukemia [36], and diffuse large B-cell lymphoma [37].

It is unknown whether there is an increased risk of having cancer in those with a gene premutation, although there is one case report of a man with lung tumor and full mutation where the tumor's origin was in a cell with premutation instead of full mutation, indicating that the neoplasia development may have been facilitated by the premutation [38]. There is a relationship between elevated mRNA and cyclic adenosine monophosphate (cAMP) response-element binding protein (CREB). The elevated mRNA is due to increased trancription [7], while CREB is involved in *FMR1 *transcriptional activity [39] and may play a role in oncogenesis by various mechanisms including gene amplification, chromosome translocation, interaction with viral oncoprotein, and inactivation of tumor supressor genes [40].

The current report of MGUS and FXTAS combined with the commonality of miRNA mechanisms in these two conditions suggests that this association may be more common than previously realized and this deserves further study. One possibility is screening for the premutation when MGUS is identified. This would be very important if FXTAS symptoms are present or if there is a family history of fragile X-associated disorders including FXPOI or fragile X syndrome or autism.

## Figures and Tables

**Table 1:** FXTAS clinical, neuroimaging, and molecular results of the patient.

Intention tremor	began at age 55
Right upper extremity	Postural tremor (+)
	Kinetic tremor (+)
Left upper extremity	Postural tremor (+)
	Kinetic tremor (+)
Handwriting problems	began at age 58
Balance problem	began at age 58
Type of gait	Ataxic and broad based
Dependent on walker	Began at age 66
Memory problems	Began at age 63
Fragile X DNA test (CGG repeat number)	98–103 (premutation range)
*FMR1 *mRNA level	2.33 ± 0.02 times normal
FXTAS diagnosis	Definite
FXTAS stage	4

**Table 2:** MGUS laboratory workup of the patient.

	Levels	Reference range	
Red cell count	3.82	4.5–5.9 M/MM3	Low
Hemoglobin	11.9	13.5–17.5 GM/DL	Low
Hematocrit	34.7	41–53%	Low
Glucose	358	70–110 mg/dL	High
Hemagobin A1C	8.5	3.9–5.9%	High
Calcium	9.5	8.6–10.5 mg/dL	Normal
Serum protein electrophoresis with immunofixation	1 g/dL IgG kappa monoclonal paraproteinemia		
Serum creatinine	1.3 mg/dL	0.6–1.2 mg/dL	Mildly elevated
GFR	55	>60	Low
IgM	17 mg/dL	37 mg/dL–50 mg/dL	Decreased
Free kappa light chain	7.81 mg/dL	0.33–1.92 mg/dL	Elevated
Lambda light chain	1.88 mg/dL	0.57–2.63 mg/dL	Normal
Kappa to lambda light chain ratio	4.15	0.26–1.65	Elevated
Beta-2 microglobulin	3.38 mcg/mL	1.8 mcg/mL	Elevated
Bone survey			No lytic or sclerotic lesions

## References

[1] Jacquemont S, Hagerman RJ, Leehey M (2003). Fragile X premutation tremor/ataxia syndrome: molecular, clinical, and neuroimaging correlates. *American Journal of Human Genetics*.

[2] Coffey SM, Cook K, Tartaglia N (2008). Expanded clinical phenotype of women with the *FMR1* premutation. *American Journal of Medical Genetics, Part A*.

[3] Adams JS, Adams PE, Nguyen D (2007). Volumetric brain changes in females with fragile X-associated tremor/ataxia syndrome (FXTAS). *Neurology*.

[4] Berry-Kravis E, Goetz CG, Leehey MA (2007). Neuropathic features in fragile X premutation carriers. *American Journal of Medical Genetics, Part A*.

[5] Rodriguez-Revenga L, Madrigal I, Pagonabarraga J (2009). Penetrance of *FMR1* premutation associated pathologies in fragile X syndrome families. *European Journal of Human Genetics*.

[6] Jacquemont S, Hagerman RJ, Leehey MA (2004). Penetrance of the fragile X-associated tremor/ataxia syndrome in a premutation carrier population. *Journal of the American Medical Association*.

[7] Tassone F, Beilina A, Carosi C (2007). Elevated *FMR1* mRNA in premutation carriers is due to increased transcription. *RNA*.

[8] Garcia-Arocena D, Hagerman PJ (2010). Advances in understanding the molecular basis of FXTAS. *Human Molecular Genetics*.

[9] Sellier C, Rau F, Liu Y (2010). Sam68 sequestration and partial loss of function are associated with splicing alterations in FXTAS patients. *EMBO Journal*.

[10] Ross-Inta C, Omanska-Klusek A, Wong S (2010). Evidence of mitochondrial dysfunction in fragile X-associated tremor/ataxia syndrome. *Biochemical Journal*.

[11] Napoli E, Ross-Inta C, Wong S (2011). Altered zinc transport disrupts mitochondrial protein processing/import in fragile X-associated tremor/ataxia syndrome. *Human Molecular Genetics*.

[12] Greco CM, Berman RF, Martin RM (2006). Neuropathology of fragile X-associated tremor/ataxia syndrome (FXTAS). *Brain*.

[13] Hashimoto R, Javan A, Tassone F, Hagerman RJ, Rivera SM (2011). A voxel-based morphometry study of grey matter loss in fragile X-associated tremor/ataxia syndrome. *Brain*.

[14] Kyle RA, Child JA, Anderson K (2003). Criteria for the classification of monoclonal gammopathies, multiple myeloma and related disorders: a report of the International Myeloma Working Group. *British Journal of Haematology*.

[15] Kyle RA, Therneau TM, Rajkumar SV (2006). Prevalence of monoclonal gammopathy of undetermined significance. *The New England Journal of Medicine*.

[16] Kyle RA, Kumar S (2009). The significance of monoclonal gammopathy of undetermined significance. *Haematologica*.

[17] Bida JP, Kyle RA, Therneau TM (2009). Disease associations with monoclonal gammopathy of undetermined significance: a population-based study of 17,398 patients. *Mayo Clinic Proceedings*.

[18] Kyle RA (1978). Monoclonal gammopathy of undetermined significance. Natural history in 241 cases. *American Journal of Medicine*.

[19] Kyle RA, Therneau TM, Rajkumar SV, Larson DR, Plevak MF, Melton LJ (2004). Long-term follow-up of 241 patients with monoclonal gammopathy of undetermined significance: the original Mayo Clinic series 25 years later. *Mayo Clinic Proceedings*.

[20] Kyle RA (1993). “Benign” monoclonal gammopathy—after 20 to 35 years of follow-up. *Mayo Clinic Proceedings*.

[21] Vachon CM, Kyle RA, Therneau TM (2009). Increased risk of monoclonal gammopathy in first-degree relatives of patients with multiple myeloma or monoclonal gammopathy of undetermined significance. *Blood*.

[22] Landgren O, Kyle RA, Hoppin JA (2009). Pesticide exposure and risk of monoclonal gammopathy of undetermined significance in the Agricultural Health Study. *Blood*.

[23] Jego G, Bataille R, Geffroy-Luseau A, Descamps G, Pellat-Deceunynck C (2006). Pathogen-associated molecular patterns are growth and survival factors for human myeloma cells through toll-like receptors. *Leukemia*.

[24] Dinarello CA (2009). Targeting the pathogenic role of interleukin 1*β* in the progression of smoldering/indolent myeloma to active disease. *Mayo Clinic Proceedings*.

[25] Bergsagel PL, Kuehl WM (2001). Chromosome translocations in multiple myeloma. *Oncogene*.

[26] Bergsagel PL, Kuehl WM (2005). Molecular pathogenesis and a consequent classification of multiple myeloma. *Journal of Clinical Oncology*.

[27] Calvo KR, Landgren O, Roccaro AM, Ghobrial IM (2011). Role of microRNAs from monoclonal gammopathy of undetermined significance to multiple myeloma. *Seminars in Hematology*.

[28] Ambros V (2004). The functions of animal microRNAs. *Nature*.

[29] Pichiorri F, Suh SS, Ladetto M (2008). MicroRNAs regulate critical genes associated with multiple myeloma pathogenesis. *Proceedings of the National Academy of Sciences of the United States of America*.

[30] Zhoua Y, Chena L, Barlogiea B (2010). High-risk myeloma is associated with global elevation of miRNAs and overexpression of EIF2C2/AGO2. *Proceedings of the National Academy of Sciences of the United States of America*.

[31] Gutiérrez NC, Sarasquete ME, Misiewicz-Krzeminska I (2010). Deregulation of microRNA expression in the different genetic subtypes of multiple myeloma and correlation with gene expression profiling. *Leukemia*.

[32] Bartel DP (2004). MicroRNAs: genomics, biogenesis, mechanism, and function. *Cell*.

[33] Sellier C, Hagerman P, Willemsen R DROSHA/DGCR8 sequestration by expanded CGG repeats leads to global micro-RNA processing alteration in FXTAS patients [abstract].

[34] Lu J, Getz G, Miska EA (2005). MicroRNA expression profiles classify human cancers. *Nature*.

[35] Jongen-Lavrencic M, Sun SM, Dijkstra MK, Valk PJM, Löwenberg B (2008). MicroRNA expression profiling in relation to the genetic heterogeneity of acute myeloid leukemia. *Blood*.

[36] Visone R, Rassenti LZ, Veronese A (2009). Karyotype-specific microRNA signature in chronic lymphocytic leukemia. *Blood*.

[37] Zhang J, Jima DD, Jacobs C (2009). Patterns of microRNA expression characterize stages of human B-cell differentiation. *Blood*.

[38] de Graaff E, Willemsen R, Zhong N (1995). Instability of the CGG repeat and expression of the *FMR1* protein in a male fragile X patient with a lung tumor. *American Journal of Human Genetics*.

[39] Smith KT, Nicholls RD, Reines D (2006). The gene encoding the fragile X RNA-binding protein is controlled by nuclear respiratory factor 2 and the CREB family of transcription factors. *Nucleic Acids Research*.

[40] Siu YT, Jin DY (2007). CREB—a real culprit in oncogenesis. *FEBS Journal*.

